# Vascular attenuation and volumetric lung iodine density in dual-layer spectral CT pulmonary angiography: a randomized controlled trial comparing three contrast doses

**DOI:** 10.1007/s00330-025-12309-2

**Published:** 2026-01-30

**Authors:** David Ferrández-Ferrández, Juan José Arenas-Jiménez, Almudena Ureña Vacas, Marina Sirera Matilla, Eloísa Feliu Rey, Víctor Marquina Arribas, Helena Trigueros Buil, Elena García-Garrigós

**Affiliations:** 1 Department of Radiology, Hospital General Universitario Dr. Balmis de Alicante, Alicante, Spain; 2 Alicante Institute for Health and Biomedical Research (ISABIAL), Alicante, Spain; 3 Department of Pathology and Surgery, Miguel Hernández University, Alicante, Spain

**Keywords:** Computed tomography angiography, Pulmonary embolism, Contrast media, Administration and dosage

## Abstract

**Objectives:**

To evaluate vascular attenuation (VA) in conventional and low-energy virtual monoenergetic images (LEVMI), volumetric lung iodine density (VID) and quality of CT pulmonary angiography (CTPA) in dual-layer detector spectral CT using three iodinated contrast medium (ICM) administration protocols.

**Materials and methods:**

A prospective randomized single-center study including patients with CTPA to rule out pulmonary embolism (PE) was performed. Examinations were randomized to one of three ICM administration protocols: A, 40 mL at 4 mL/s; B, 30 mL at 3 mL/s; and C, 20 mL of ICM diluted with 20 mL of saline at 4 mL/s. Two radiologists evaluated the presence of PE, VA in conventional images and LEVMI, lung VID, perfusion defects detection, and quality of Z-effective maps. Statistical comparisons were performed between protocols.

**Results:**

Fifty patients were randomized to each protocol. In conventional images, VA in pulmonary arteries was above 200 HU in more than 90% in protocols A and B, but only in 70% in protocol C. VA increased in LEVMI, with a minimum value of 269 HU. Differences in pulmonary VA with protocol C were statistically significant. At LEVMI, aortic attenuation was above 100 HU in most examinations. Protocol C presented the worst quality of iodine map and the lowest VID; however, it detected perfusion defects in all PE cases.

**Conclusion:**

The use of LEVMI provides diagnostic VA levels in pulmonary arteries in all the protocols, and a minimum aortic enhancement in most cases. Even the lowest ICM dose maintains diagnostic iodine maps, although with lower quality and VID.

**Key Points:**

***Question*** *Do low doses of iodinated contrast medium for spectral CT pulmonary angiography achieve diagnostic vascular attenuation, and do they allow detection of perfusion defects in pulmonary embolism?*

***Findings*** *All three protocols achieved diagnostic pulmonary artery attenuation in low-energy virtual monoenergetic images and detected perfusion defects in all pulmonary embolism cases*.

***Clinical relevance*** *Spectral CT pulmonary angiography enables diagnostic pulmonary vascular enhancement and reliable perfusion defect detection with reduced contrast material doses, supporting safer and more efficient pulmonary embolism imaging protocols*.

**Graphical Abstract:**

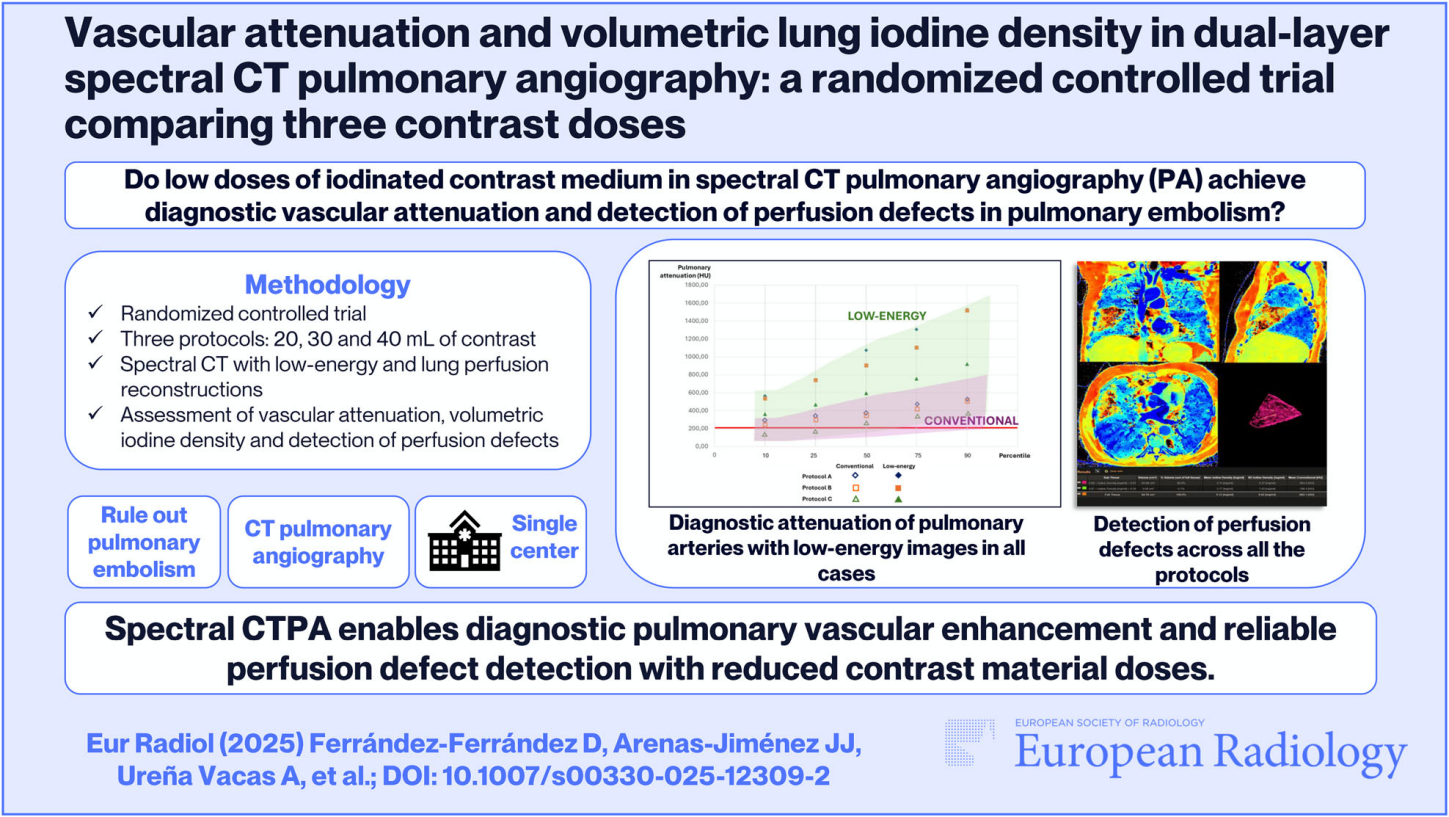

## Introduction

CT pulmonary angiography (CTPA) is the primary diagnostic imaging tool for acute pulmonary embolism (PE) [1]. Both imaging acquisition parameters and contrast medium administration protocols may vary according to several factors, mainly the technical resources available, patient characteristics and clinical requirements [2], with a broad range of radiation and contrast media doses being used across different institutions. Very low iodinated contrast medium (ICM) protocols have been shown to be feasible, with total iodine doses in the range of 6–12 g and iodine delivery rates from 0.8 to 1.6 gI/s [3–9]. The routine use of “as low a contrast medium dose as reasonably achievable” should be desirable for patient’s safety, economy, environment, and even for facing potential contrast medium shortage [10–12].

State-of-the-art CT scanners dramatically reduce scanning time, and multienergy CT permits us to obtain higher vascular attenuation (VA) by using low-energy virtual monoenergetic images (LEVMI), and to assess lung perfusion by analyzing iodine density and Z-effective maps. This allows the detection of perfusion defects due to acute PE [13–17]. Using these technologies, there are varied possibilities for ICM protocols and scanning that allow lowering from the standard routine protocol of 80–100 mL suggested by a recent clinical consensus statement [2] to 20–30 mL or even less [4, 6, 8, 18]. LEVMI provides sufficient attenuation of pulmonary arteries in non-diagnostic conventional images [19], even at low injection rates [20]. However, the impact of these lower doses on the quality of information on lung perfusion obtained from spectral CT has not been properly addressed. Whether diagnostic perfusion maps are achievable with lower doses of contrast medium is an open question.

We aimed to compare three different protocols of ICM administration with 20, 30 and 40 mL for CTPA performed in a dual-layer detector spectral CT (DLDSCT) scanner by evaluating VA in conventional and low-energy images, calculating volumetric iodine density (VID) of normal lung and analyzing the detectability of perfusion defects. Our hypothesis was that the lower dose provided diagnostic contrast enhancement of the pulmonary arteries in LEVMI and adequate iodine density and perfusion information.

## Materials and methods

This prospective randomized control trial was approved by the institutional review board of Hospital General Universitario Dr. Balmis in Alicante (Spain) and registered at the Clinical Trials Information System of the European Medicines Agency with the number 2024-515246-17-00 approved on 19 October 2024. All participants gave written informed consent.

### Sample size estimation and randomization

Details about sample size estimation and method for randomization are described in the corresponding section of Supplementary Material.

### Study population

Between 11 November 2024 and 1 August 2025, all the patients being admitted to the DLDSCT in Hospital General Universitario Dr. Balmis de Alicante for a CTPA to rule out acute PE were considered candidates for the study.

The inclusion criteria comprised being at least 18 years old with clinical suspicion of acute PE and an estimated glomerular filtration rate by the CKD-EPI 2021 equation greater than 30 mL/min/1.73 m^2^. The exclusion criteria are detailed in the study population section of Supplementary Material, among them weighing less than 50 or more than 100 kg.

### Imaging acquisition, contrast administration protocols and radiation dose

All the examinations were performed using a DLDSCT (CT 7500, Philips Healthcare). The acquisition parameters were as follows: tube voltage 120 kVp, collimation 128 × 0.625 mm, rotation time 0.27 s, automatic tube current adapted to the patient size (DoseRight Index 17, Philips Healthcare; reference tube current 50–300 mAs). For initiating the scan, the bolus tracking technique was used, with acquisition initiating 6 s after reaching an attenuation of 110 HU in the pulmonary artery trunk. Images were reconstructed in axial orientation with a slice thickness of 1 mm every 0.5 mm. More information about acquisition and reconstruction parameters is detailed in the Supplementary Material.

All patients received an intravenous bolus of ICM with 400 mg iodine/mL (Iomeron 400, Bracco Imaging) through a peripheral vein. Due to a stock shortage of this specific iodine concentration, the inclusion period suffered from some interruptions.

Contrast administration protocols were as follows:

- Protocol A: 40 mL of ICM, followed by 40 mL of saline solution, both at 4 mL/s (16 g of iodine at a iodine delivery rate of 1.6 gI/s);
- Protocol B: 30 mL of ICM followed by 40 mL of saline solution, both at 3 mL/s (12 gI at 1.2 gI/s);
- Protocol C: 40 mL of a 50% saline and ICM solution, followed by 40 mL of saline, both at 4 mL/s, thus constituting 20 mL of contrast media (8 gI at 0.8 gI/s).

The dose length product (DLP) in mGy and volume CT dose index (CTDIvol) in mGy.cm were recorded for each patient.

### Patient characteristics and PE diagnosis

The patient’s age, sex, height and weight were recorded, and body mass index was calculated. For the diagnosis of PE, it was considered the unequivocal presence of pulmonary artery filling defects assessed by two experienced radiologists, or in the case of discrepancies between them, by consensus with a third experienced radiologist.

### Image analysis

Two thoracic radiologists (A.U.V., J.J.A.J., with 8 and 27 years of experience in thoracic CT interpretation), blinded to the contrast administration protocol, independently reviewed CT images.

The method for diagnosing PE and its classification as central or peripheral is described in the Supplementary Material.

### Quantitative analysis

Vascular attenuation was measured in the pulmonary trunk and right and left pulmonary and interlobar arteries. Mean pulmonary attenuation and contrast-to-noise ratio (CNR) and signal-to-noise ratio (SNR) were measured at the pulmonary arteries in the conventional and low-energy 45 keV images. Ascending aorta attenuation was also measured. One radiologist calculated the absolute VID in mgI/mL of both lungs by using “Volumetric quantification” in Intellispace Portal (Philips Healthcare) according to the method described by Kroeger et al [21]. Basically, a volume of the normal lung in a 3D reconstruction was selected in each lung. Afterward, taking as the reference the iodine density in the left atrium, the vascular compartment was segmented and separated from the lung, whose iodine density by mL was calculated. The same method was performed for calculating the VID of the hypoperfused lung in the case of PE.

Details about the method for all these measurements are described in Supplementary Material in the quantitative analysis heading.

### Qualitative analysis

Both readers subjectively assessed the quality of Z-effective images to evaluate the perfusion defects and the presence of artifacts in those images according to the Likert scales described in Supplementary Material.

### Statistical analysis

Normality of distributions was assessed using histograms and the Kolmogorov-Smirnov test. Continuous variables were expressed as means ± standard deviation in the case of normal distribution or medians (interquartile range, IQR, Q1-Q3) otherwise. Categorical variables were expressed as numbers and percentages. For continuous variables, comparisons between the three protocols on the whole were performed using the analysis of variance (ANOVA) in case of normal distribution, and post hoc analysis was performed with Tukey’s honestly significant difference. Continuous variables with nonparametric distribution were assessed using the Kruskal–Wallis test and Dunn–Bonferroni corrected post hoc analysis. Differences between the subgroups for categorical variables were analyzed with Chi-square. Pearson’s correlation coefficient and Spearman’s rank correlation between weight and body mass index, with mean attenuation of the pulmonary arteries, and DLP and CTDIvol, respectively, were calculated.

Cohen’s Kappa was calculated for interobserver agreement for qualitative assessment and Pearson’s correlation coefficient for quantitative assessments.

Statistical testing was conducted at the two-tailed α-level of 0.05.

Data were analyzed using SPSS version 29.0.

## Results

### Patient characteristics

**Fig. 1:**
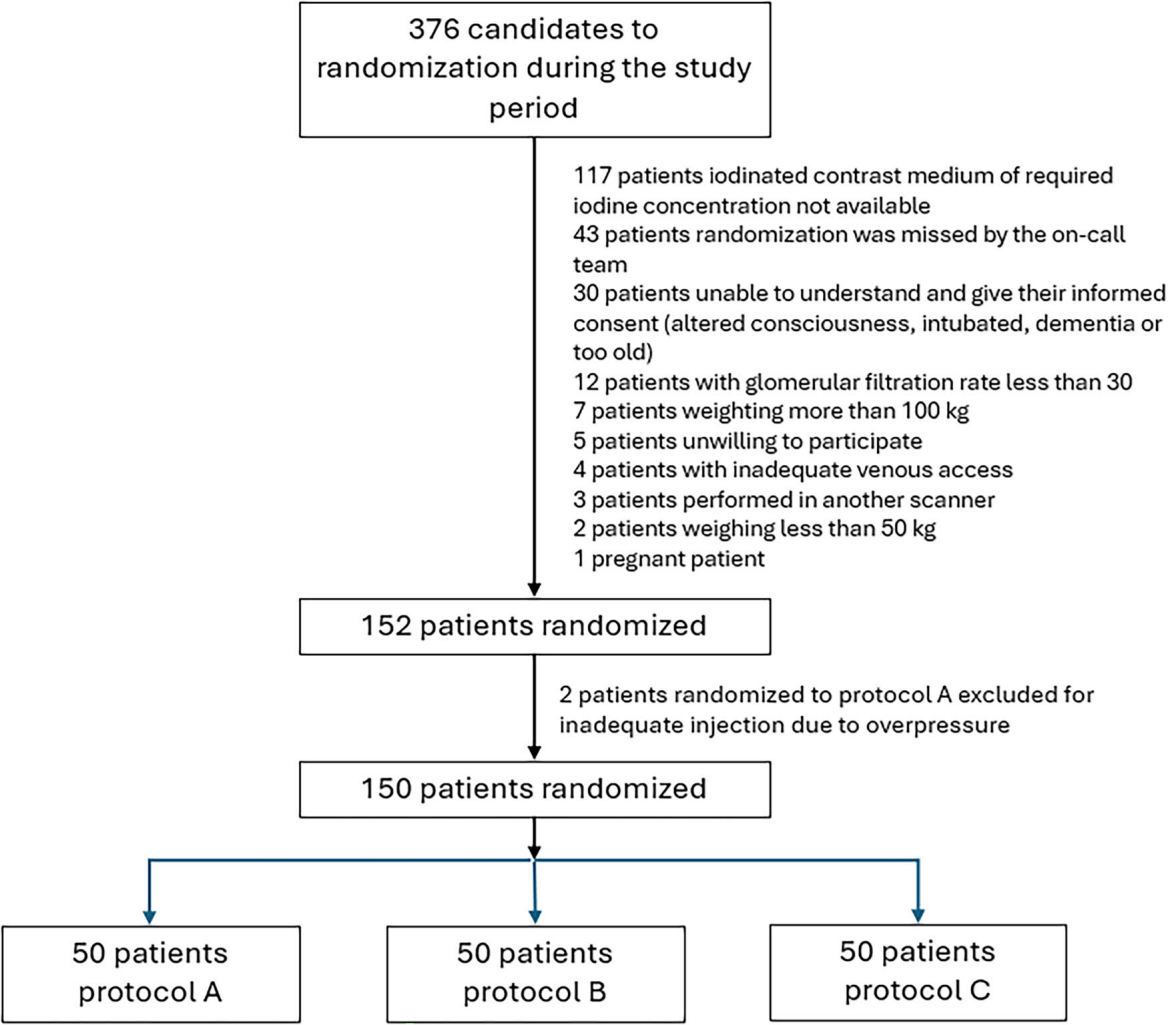
Flowchart showing patient inclusion process

Figure 1 shows the flowchart of patients included. Two patients initially randomized to protocol A were not included because contrast administration could not be completed according to the protocol due to overpressure warning during injection. In the end, 50 patients were included in each protocol. Patient characteristics are shown in Table 1.

**Table 1:** Patient characteristics

	Protocol A *n* = 50	Protocol B *n* = 50	Protocol C *n* = 50	*p*-value
Age (years)	62.5 ± 15.5	68.5 ± 16.4	59.7 ± 16.7	0.022^a^
Sex (male/female)	22/28	20/30	25/25	0.599
Weight (kg)	74.6 ± 13.4	71.2 ± 12.7	72.7 ± 12.5	0.421
BMI (kg/m^2^)	27.4 ± 4.7	26.2 ± 3.8	25.9 ± 3.5	0.173

^a^ Differences A vs B *p* = 0.149; A vs C *p* = 0.668; B vs C *p* = 0.019

### Diagnosis of pulmonary embolism

**Fig. 2:**
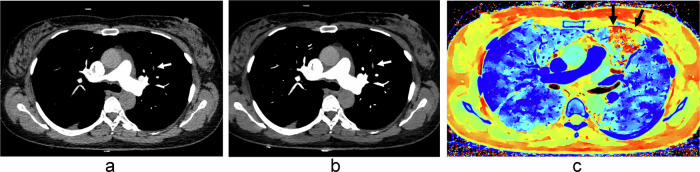
60-year-old woman with suspected acute pulmonary embolism. CT pulmonary angiography with contrast administration using protocol C shows a subsegmental pulmonary embolism in a branch of the artery for the anterior segment of the left upper lobe (white arrows in **a** and **b**). One reader missed the clot in conventional images (**a**) and 45 keV virtual monoenergetic image (**b**) but detected the perfusion defect in the Z-effective map (black arrows in **c**). The other reader diagnosed pulmonary embolism in all three images

PE was diagnosed in 37 patients in total (24.7%), 24 of them (64.9%) were in a central location. The number of patients diagnosed with PE at each protocol, their location and the number of cases diagnosed by image modality are shown in Table 2. There were only 3 discordances, two of which were isolated subsegmental PE missed in conventional images by one reader each, and the third one was one of these isolated subsegmental PE that was also missed by the same reader in LEVMI (Fig. 2). In both cases, a perfusion defect was detected by the reader, and PE was diagnosed in the consensus reading.

**Table 2:** Diagnosis of pulmonary embolism

	Protocol A *n* = 50	Protocol B *n* = 50	Protocol C *n* = 50	Total number of PE by consensus
Final consensus diagnosis of PE	9	13	15	37
Central	8/8	7/7	9/9	24
Peripheral	1/1	6/6	6/6	13
Conventional images	9/9	13/12	14/15	37
Low-energy 45-keV images	9/9	13/12	15/15	37
Z-effective map images	9/9	13/13	15/15	37

Data are the number of patients diagnosed with pulmonary embolism by each reader (reader 1/reader 2)

*PE* pulmonary embolism

### Vascular attenuation, signal-to-noise and contrast-to-noise ratio

**Fig. 3:**
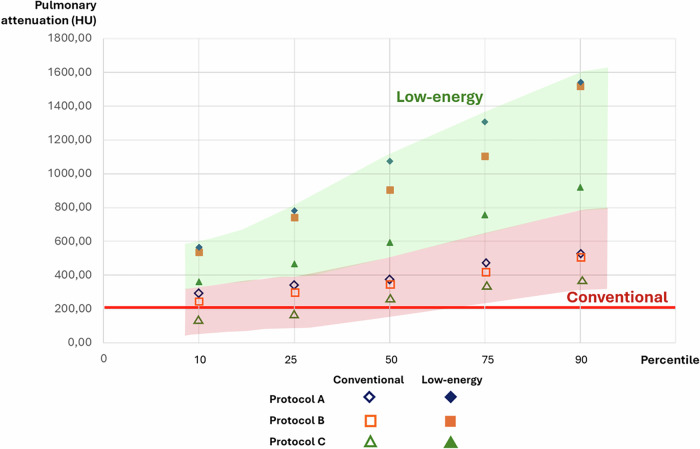
Graphic representation of the percentile distribution of mean pulmonary artery attenuation for both readers in conventional and at 45 keV virtual monoenergetic images by protocol. The red line represents the diagnostic level reference at 200 HU

Values of VA, SNR and CNR of both readers by protocol are shown in Table 3, and Supplementary Tables S1 and S2. At all levels of the pulmonary artery tree, in both conventional and low-energy measurements, VA was statistically significantly lower in protocol C compared with protocols A and B. In all cases, mean pulmonary VA was higher in protocol A, although differences with protocol B were not statistically significant. When analyzing the minimum value and percentile distributions (Supplementary Tables S3, S4) in conventional images, protocol C showed the minimum values. Fifteen out of 50 (30%) patients in protocol C showed pulmonary arterial attenuation below 200 HU. In LEVMI, every measurement of VA of pulmonary arteries was higher than 200 HU, including those in protocol C (Fig. 3).

**Table 3:** Summary of mean vascular attenuation and minimum values at the pulmonary artery and ascending aorta and contrast-to-noise and signal-to-noise ratio in conventional and low-energy by protocol

		Protocol A		Protocol B		Protocol C		*p*-values^a^		
		Mean ± standard deviation	Minimum	Mean ± standard deviation	Minimum	Mean ± standard deviation	Minimum	*p*-value A vs B	*p*-value A vs C	*p*-value B vs C
Mean pulmonary artery^b^	Reader 1	373.2 ± 105.2	180	352.5 ± 103.5	161	246.5 ± 69.7	118	0.278	0.000	0.000
	Reader 2	372.9 ± 104.5	179	351.8 ± 103.1	164	244.8 ± 69.5	109	0.266	0.000	0.000
Mean pulmonary artery at LEVMI^b^	Reader 1	1056.4 ± 353.1	457	957.6 ± 329.1	397	618.7 ± 204.1	256	0.108	0.000	0.000
	Reader 2	1054.4 ± 351.7	460	972.1 ± 352.7	390	621.3 ± 203.4	266	0.192	0.000	0.000
Ascending aorta	Reader 1	208.9 ± 88.8	71	170.7 ± 65.8	52	145.8 ± 56.3	48	0.011	0.000	0.065
	Reader 2	212.3 ± 92.1	71	174.6 ± 65.7	49	146.2 ± 57.2	50	0.014	0.000	0.053
Ascending aorta at LEVMI	Reader 1	529.0 ± 274.4	91	403.6 ± 188.7	63	340.1 ± 165.9	75	0.006	0.000	0.127
	Reader 2	523.8 ± 278.8	100	404.2 ± 187.6	74	337.2 ± 166.7	80	0.006	0.000	0.119
Contrast-to-noise ratio	Reader 1	12.4 ± 4.2	4.3	12.9 ± 4.7	4.9	9.4 ± 3.6	2.5	0.845	0.001	0.000
	Reader 2	13.0 ± 4.3	5.1	13.0 ± 4.9	5.2	9.2 ± 3.3	2.2	1.000	0.000	0.000
Contrast-to-noise ratio at LEVMI	Reader 1	35.0 ± 15.8	10.1	34.0 ± 13.7	9.9	23.1 ± 8.6	5.7	0.708	0.000	0.000
	Reader 2	34.5 ± 14.5	12.2	36.6 ± 25.5	10.2	23.2 ± 8.5	5.1	0.548	0.002	0.000
Signal-to-noise ratio	Reader 1	15.4 ± 4.5	6.8	15.4 ± 5.1	5.1	12.1 ± 3.5	4.6	0.743	0.011	0.001
	Reader 2	14.6 ± 4.3	5.9	15.3 ± 4.9	7.3	12.1 ± 3.8	4.1	0.999	0.001	0.001
Signal-to-noise ratio at LEVMI	Reader 1	38.0 ± 16.1	12.7	37.1 ± 14.0	12.4	26.6 ± 8.9	8.4	0.735	0.000	0.000
	Reader 2	37.5 ± 14.9	15.2	39.7 ± 25.9	10.9	26.9 ± 8.7	8.0	0.534	0.000	0.000

*LEVMI* low-energy virtual monoenergetic images at 45 keV

^a^ Overall differences between protocols statistically significant (*p* < 0.001) for all measurements

^b^ Mean pulmonary artery attenuation was calculated as the mean value of the pulmonary trunk, right and left pulmonary arteries and right and left interlobar arteries

Pearson’s correlation coefficients between weight and body mass index (BMI) with pulmonary attenuation are shown in Table S5 and Fig. S6. Body weight showed a higher correlation than BMI, particularly in protocol B, with a statistically significant negative correlation (−0.458; *p* < 0.01).

Mean values of VA in the ascending aorta decreased progressively from protocol A to B and C, with differences statistically significant between protocols A and B for conventional and low-energy images. Four patients in protocol A and 8 in protocols B and C, respectively, showed at least one measurement lower than 100 HU at the aorta in the conventional images (Supplementary Table S6). However, in LEVMI, it occurred in only 4 cases. In all these cases, a post hoc analysis of the examinations revealed radiologic findings consistent with heart failure. In 2 cases (in protocols B and C, respectively), a relevant aortic disease was detected by both readers in the CTPA (Supplementary Figs. S7, S8).

SNR and CNR were statistically significantly lower in protocol C compared with A and B, which showed very similar results. For both parameters, conventional images showed lower values compared with LEVMI.

All of these quantitative measurements showed a strong interobserver correlation above 0.9.

### Volumetric iodine density of normal lung and perfusion defects

VID could not be calculated in 6 patients due to extensive lung opacities, severe emphysema or combined parenchymal hypoperfusion and lung opacities. Median VID was statistically different between groups, as shown in Table 4, with lower values in protocol C. However, attenuation of the hypoperfused lung did not show statistically significant differences (*p* = 0.20). In all cases, the maximum value of hypoperfused parenchyma was lower than the corresponding minimum value of normal lungs for each protocol.

**Table 4:** Volumetric iodine density of normal and hypoperfused lung by protocol and their minimum and maximum value, respectively

	Normal lungs		Hypoperfused lung	
	Minimum	Median (IQR)^a^	Maximum	Median (IQR)^b^
Protocol A *n* = 47/8	0.41	0.98 (0.85–1.33)	0.33	0.13 (0.08–0.24)
Protocol B *n* = 48/11	0.34	0.79 (0.56–0.97)	0.21	0.09 (0.08–0.12)
Protocol C *n* = 49/13	0.30	0.50 (0.40–0.65)	0.19	0.09 (0.06–0.15)

Values of “*n*” are the number of patients with VID measured in normal lungs/number of patients with VID measured in hypoperfused lung

^a^ Differences between all the groups protocols, *p* < 0.001; A vs B *p* = 0.017; A vs C *p* < 0.001; B vs C *p* < 0.001

^b^ Differences between protocols, *p* = 0.205

### Qualitative evaluation

For statistical analysis, grading of the quality of Z-effective images for evaluating perfusion defects was divided into grade 1, encompassing “excellent” and “adequate” categories, and grade 2, including the rest of the lower quality categories. Results are shown in Table 5. Protocol C showed the highest proportion of low-quality ratings that accounted for 82% of all the readings in that protocol, compared with 33% and 51% in protocols A and B, respectively.

**Table 5:** Subjective rating of the quality of Z-effective maps for assessment of perfusion defects and grading of artifacts

	Score	Protocol A *n* = 50	Protocol B *n* = 50	Protocol C *n* = 50	*p*-value
Quality of maps (reader 1)	1	32 (64%)	24 (48%)	13 (26%)	0.001
	2	18 (36%)	26 (52%)	37 (74%)	
Quality of maps (reader 2)	1	35 (70%)	25 (50%)	5 (10%)	0.000
	2	15 (30%)	25 (50%)	45 (90%)	
Artifacts (reader 1)	1	40 (80%)	44 (88%)	42 (84%)	0.551
	2	10 (20%)	6 (12%)	8 (16%)	
Artifacts (reader 2)	1	42 (84%)	47 (94%)	43 (86%)	0.266
	2	8 (16%)	3 (6%)	7 (14%)	

Quality of maps: score 1: “excellent” and “adequate” categories, and score 2: “acceptable,” “poor,” and “unusable”

Artifacts: score 1: “absence of artifacts,” “mild that do not interfere” or “moderate that slightly affect reading,” and score 2: “moderate that clearly interfere with reading,” and “severe artifacts that prevent reading”

Artifacts were categorized as grade 2, corresponding to significant artifacts (scores 4 and 5), and grade 1 (scores 1 to 3), corresponding to artifacts that did not affect reading (Table 5). Protocol B showed a lower proportion of significant artifacts (9%, compared with 15% in protocol C and 18% in A). However, differences were not statistically significant.

For both readings, there was substantial agreement between the two observers (kappa = 0.677).

### Estimated radiation dose

There were no statistically significant differences in DLP and CTDIvol between protocols, as shown in Supplementary Table S7. Spearman’s rank correlation between DLP and CTDIvol with weight and BMI was greater for weight, which reached 0.810 for DLP (Supplementary Table S8).

## Discussion

In this prospective randomized controlled trial, we demonstrate that by using LEVMI, all three protocols of ICM administration in CTPA provided VA in the pulmonary arteries within the diagnostic range. Protocols A and B were similar in terms of VA, quality of perfusion maps, and aortic enhancement. Although perfusion defects were visible at Z-effective maps in all three protocols, VID and the qualitative evaluation of these maps were significantly lower in the 20 mL protocol.

Although there is no “one size fits all” in contrast administration protocols, it is necessary to determine how some parameters influence the quality of the studies using spectral CT. It is well established that LEVMI provides higher attenuation [22], which enables diagnostic examinations with the use of low-contrast medium doses [5] and helps salvage otherwise non-diagnostic studies[19]. There is also evidence that information about iodine distribution obtained from spectral CT may be used as a surrogate for perfusion [23] and that it adds an incremental benefit in the diagnosis of PE by easing the detection of perfusion defects due to thrombi that may be missed in anatomical images [24, 25]. However, the questions about how these low-contrast doses affect the quality of information from iodine maps, their possible limitations, and their practical utility are unresolved in the literature. The iodine doses of 8 and 12 g, and iodine delivery rates of 0.8 and 1.2 gI/s in protocols C and B, respectively, were taken from the literature describing the use of low doses of IMC for CTPA [3–9].

We found a high incidence of PE in our population, with most cases detected using conventional images and a few discrepancies at a patient level, which were limited to subsegmental emboli detected with LEVMI. In a single case, and by only one reader, PE was diagnosed after recognition of a perfusion defect, an incidence of 0.7%, which was slightly lower than that reported by Weidman et al [25], who found it to be 1%.

Even with the lowest dose protocol, the theoretical diagnostic attenuation threshold of 211 HU proposed by Wittram [26] was surpassed by LEVMI across all the anatomic levels of the pulmonary arteries. This finding differs from the results by Kristiansen et al [5], who used an iodine delivery rate of about 0.95 gl/s (between those of our protocols B and C) and still reported some vascular attenuation values below this threshold. In conventional images, protocol C was insufficient, with 30% below 200 HU, although in protocols A and B, there were also outliers, with some cases with VA below these values. Thus, if we consider VA only, low-energy images guarantee a diagnostic examination across all protocols with better results in protocols A and B. The latter protocol (B), by injecting at 3 mL/s, has the advantage of admitting the use of catheters with lower gravity flow rates and poorer venous accesses [25], while also providing 25% contrast saving compared with protocol A.

Each manufacturer has its own technology and software for perfusion evaluation in spectral CT. All of them are based on the information obtained from iodine distribution, which serves as a surrogate for perfusion [13, 15]. In at least one study [17], the Z-effective derived maps have been reported to outperform iodine-based maps for detecting perfusion defects. In our experience with the platform used in this study, good-quality Z-effective maps are a rapid and accurate way to detect perfusion defects in combination with the evaluation of iodine density and conventional vascular and lung images. Thus, evaluation of their quality across the different contrast doses was an important goal of our study. On subjective assessment of the Z-effective maps, protocol C demonstrated the lowest quality, with 82% considered to have significant limitations for evaluating potential perfusion defects. This finding is consistent with prior work showing that higher iodine delivery rates of ICM provided better perfusion-map images of the lung compared with lower rates [27]. However, more objective methods for perfusion-map quality assessment remain desirable [28]. In this regard, assessment of VID allows a quantification of iodine density in the lungs, similar to that obtained by VA measurements in the vessels. Yasaka et al [29] analyzed the impact of inspiratory depths on the iodine density of the lung; however, their contrast dose and administration protocol were different from those in our study, and measurements were based on ROI in the axial plane. VID has been evaluated by one investigation group in acute PE [30], pulmonary hypertension [31], and chronic PE [21], but no study has evaluated the impact of different doses of contrast on VID. Unlike these studies, which calculated normalized VID for the whole lungs, our methodology used an absolute volumetric ROI that excluded artifacts and parenchymal abnormalities, allowing direct comparison among protocols. We found a significant decrease in the VID when lowering the contrast dose. Although hypoperfused regions remained visually apparent in all PE cases, differences were more pronounced with protocols A and B. No established VID thresholds exist to determine whether perfusion defects can be reliably detected. Using an analogous approach to that proposed by Wittram et al [26] for determining the minimum VA required for PE diagnosis, we estimated a theoretical minimum VID for detecting perfusion defects by adding three times the interquartile range of hypoperfused lung to its median value. For protocol A, this yielded 0.61 mgI/mL (13 + (3 × 0.16)). Median VID in protocol C fell below this level. Low-quality perfusion maps, together with these low VID values, make protocol C insufficient for effectively assessing perfusion.

Finally, aortic enhancement was also evaluated. Although not the primary role of CTPA, achieving acceptable aortic attenuation may be helpful in the clinical setting. For this reason, the contrast injection duration of 10 s used in our study was chosen by considering the pulmonary to aortic transit time [32–34] to allow the contrast bolus to arrive in the aorta. By considering 100 HU as a “minimal valuable attenuation” in the aorta, again, low-energy images achieved that level in most cases, with the unique exception of 4 patients with heart failure. Published data on aortic attenuation achieved in CTPA examinations are variable, with many studies investigating low-contrast dose CTPA that did not report aortic attenuation [3, 5–9]. Other studies reporting higher aortic attenuation than ours lacked key protocol parameters, such as triggering delay, ROI placement or even the contrast dose, limiting interpretation of discrepant results [19, 35]. When PE is the only clinical suspicion, we consider that the use of any of these three protocols provides at least minimal information regarding the aorta. Notably, two cases of significant aortic disease were diagnosed in our cohort.

Our study had some limitations. First, due to the study design, we did not take into consideration patient characteristics, which may be used for an individualized approach in each patient. Although tailoring of contrast and radiation dose to the patient should be desirable, our results are valuable in that they provide information about the strengths and limitations of each protocol in a general population, thus contributing to applying that information when individualizing protocols. Second, the VID was measured in a volumetric ROI instead of the whole lung. This choice was intentional, as whole lung measurements may be affected by artifacts and parenchymal abnormalities; limiting the ROI to normal lung parenchyma allowed more consistent comparisons. Third, the greater proportion of central PE may be a limitation for generalization of the suitability of each protocol for diagnosing peripheral PE, since they were underrepresented in our series.

In conclusion, in CTPA performed in DLDSCT, the use of LEVMI provided VA at diagnostic levels and allowed detection of perfusion defects at Z-effective maps in all three protocols investigated. Based on our results, the protocol with 30 mL at 3 mL/s is an alternative to the standard 40 mL at 4 mL/s in all the parameters studied, and it can be recommended as the standard protocol for routine practice with this scanner. Contrarily, the protocol with 20 mL showed the lowest attenuation and volumetric iodine density values and a significant rate of poor-quality maps.

## Supplementary information

ELECTRONIC SUPPLEMENTARY MATERIAL

## Abbreviations

BMI: Body mass index
CNR: Contrast-to-noise ratio
CTDIvol: Volume CT dose index
CTPA: CT pulmonary angiography
DLDSCT: Dual-layer detector spectral CT
DLP: Dose length product
ICM: Iodinated contrast medium
LEVMI: Low-energy virtual monoenergetic images
PE: Pulmonary embolism
SNR: Signal-to-noise ratio
VA: Vascular attenuation
VID: Volumetric iodine density
