# Supplementary material for: Vascular attenuation and volumetric lung iodine density in dual-layer spectral CT pulmonary angiography: a randomized controlled trial comparing three contrast doses
Source: Eur Radiol. 2026 Jan 30;36(6):4801–11. doi: 10.1007/s00330-025-12309-2 (PMC13212625; doi:10.1007/s00330-025-12309-2)
Supplement: Supplementary file 1 — ELECTRONIC SUPPLEMENTARY MATERIAL [file 330_2025_12309_MOESM1_ESM.pdf]

Vascular attenuation and volumetric lung iodine density in dual-layer spectral CT pulmonary angiography: a randomized controlled trial comparing three contrast doses.

ELECTRONIC SUPLEMMENTARY MATERIAL

MATERIALS AND METHODS

Sample size estimation and randomization

Differences between groups in vascular attenuation were expected. For that reason, broad limits were presumed for calculating the sample size. With a power of 90% and type I error rate, α = 5%, admitting a standard deviation of outcome of 50 and a non-inferiority limit of 30, the sample size required for each group was 48. Thus, it was decided to randomize 150 patients, 50 in each group.
A list of random numbers for each protocol was created. Patients fulfilling the inclusion criteria and willing to participate were enrolled after signing agreement to participate. They were assigned to the correspondent protocol by the attendant team. However, they were only fully included in the study when the examination was accomplished according to the corresponding protocol, without technical deviations during contrast administration or CT acquisition. Otherwise, the patient was excluded from the study, and the random number was assigned to another patient. The patients and radiologists assessing outcomes were blinded to the randomization.

Study population

The exclusion criteria were allergy or hypersensitivity to ICM, pregnancy, refusal of the patient to participate in the study, inability to give the informed consent, weight less than 50 or more than 100 kg, or the inability to administer the contrast due to problems with venous access, with the injection pump, central line, etc.

Imaging acquisition and contrast administration protocols

Scan included the whole chest and were performed in a craniocaudal direction during a midinspiratory breath-hold. For all reconstructions, a dedicated spectral reconstruction algorithm with a soft tissue kernel was used (Spectral, B, Philips Healthcare). Matrix was set to 512 × 512. The spectral base image (SBI) data were automatically reconstructed and reviewed using the workstation (IntelliSpace Portal 10.0, Philips Healthcare). Multiparameter spectral images were retrospectively generated from the SBI. The conventional images, monoenergetic 45-keV and the Z-effective maps were reviewed in a multiplanar mode, with possibility for window adjusting. For the diagnosis of perfusion defects in PE and for tracing the volume for calculating volumetric iodine density, the readers could review the angiographic, lung window and iodine density map images.

Image analysis

A diagnosis of positive or negative for the presence of PE was established at a patient level, firstly based only on the conventional images, followed by the evaluation of LEVMI at 45 keV. PE was defined as low-attenuation filling defects within the pulmonary arteries. A third reading consisted of the evaluation at the workstation of the Z-effective, together with the whole spectral information, including iodine density, conventional and low-energy images. Finally, a definitive diagnosis of PE resolving the discrepancies between readers and reconstructions was established by both readers and a third experienced radiologist. Thus, diagnosis was established at four steps: conventional images, low-energy images, Z-effective, and consensus diagnosis. PE was classified as central if located in either the pulmonary trunk, main pulmonary artery, or lobar pulmonary artery, and peripheral, when limited to segmental or subsegmental arteries.

Quantitative analysis

By placing a circular region of interest (ROI) including at least two thirds of the vessel diameter in the center of the vessel in the axial plane, mean pulmonary attenuation in Hounsfield Units (HU) was measured at five levels: the pulmonary trunk (PT), and bilaterally in the main and interlobar arteries (Supplementary Figure S1). Attenuation of the ascending aorta was also recorded in the same section as the PT. The same measures were performed in the conventional and low-energy 45 keV images. The mean of all the five measurements of the pulmonary arteries was calculated and served as a reference for simplification when presenting the results. Standard deviation (SD) of the measurement at the pulmonary trunk was used as the noise reference. The paravertebral muscle was also measured with approximately 1 cm^2 ROI and used as a reference tissue for calculating the contrast-to-noise ratio (CNR) as the mean attenuation of the PT minus mean muscle attenuation divided by the image noise (SD at the PT). If fatty infiltration avoided its adequate measurement, the subscapular or the teres major muscle was used as a reference. Signal-to-noise ratio (SNR) was calculated as mean attenuation of the PT divided by the image noise of the PT, that is, the SD of the measurement at that level. All these measurements were performed independently by both radiologists, and they were expressed as HU.

The calculation of the absolute volumetric iodine density (VID) in mg/mL of both lungs was performed by using “Volumetric quantification” in Intellispace Portal (Philips Healthcare). A roughly spherical volume of the lung in a 3D reconstruction was selected, including at least approximately 300 cm^3 of normal lung. Delineation of the volume was performed by reviewing lung window on conventional images, Z-effective and iodine density images to ascertain the lung was normal and no perfusion defect or artifact were included in the volumetric ROI. For eliminating the

iodine density attributable to the vessel compartment, according to Kroeger et al. [21], all voxels with an iodine density greater than half or the iodine density measured in a ROI in the left atrium were separated from the voxels with lower iodine density. The latter was considered the lung compartment, and its iodine density expressed in mg/mL was registered as the VID of normal lungs (Supplementary Figure S2). A mean of VID of both lungs was regarded as the VID for that examination. In patients showing perfusion defects, one perfusion defect was chosen and delineated in a multiplanar reconstruction in Z-effective images (Supplementary Figure S3). Vascular compartment was eliminated as described for normal lung, and the remaining tissue was considered the hypoperfused lung, whose VID was measured.

Qualitative analysis

For the quality of Z-effective maps, the Likert scale was: 1, excellent, diffusely homogeneous parenchyma; 2, adequate, some non-significant areas of heterogeneity ; 3, acceptable, with some areas of significant heterogeneity of the map; 4, poor quality, with major limitation to evaluate possible areas of hypoperfusion; and 5, unusable due to extensive heterogeneity of the map precluding evaluation of potential defects. Artifacts were classified as 1, absence of artifacts; 2, mild that do not interfere; 3, moderate that slightly affect reading; 4, moderate that clearly interfere with reading; and 5, severe artifacts that prevent reading. Representative images of these scores are shown in Supplementary Figures S4 and S5.

Supplementary Figures Legends

Figure S1. 71-year-old woman with suspected pulmonary embolism. Reference sections for assessment of vascular attenuation. *a* to *e* correspond to conventional images. In *a*, red circle shows the region of interest (ROI) in the pulmonary trunk and the yellow one in the ascending aorta at the same level. Red circles are ROI at the right pulmonary artery (*b*), left pulmonary artery (*c*), left interlobar artery (*d*), and right interlobar artery (*e*). In *f*, at the same level as "*a*" virtual monoenergetic 45 keV image shows an increased attenuation, from 449 HU in the pulmonary trunk and 101 HU in the aorta, to 1265 and 198 HU, respectively.

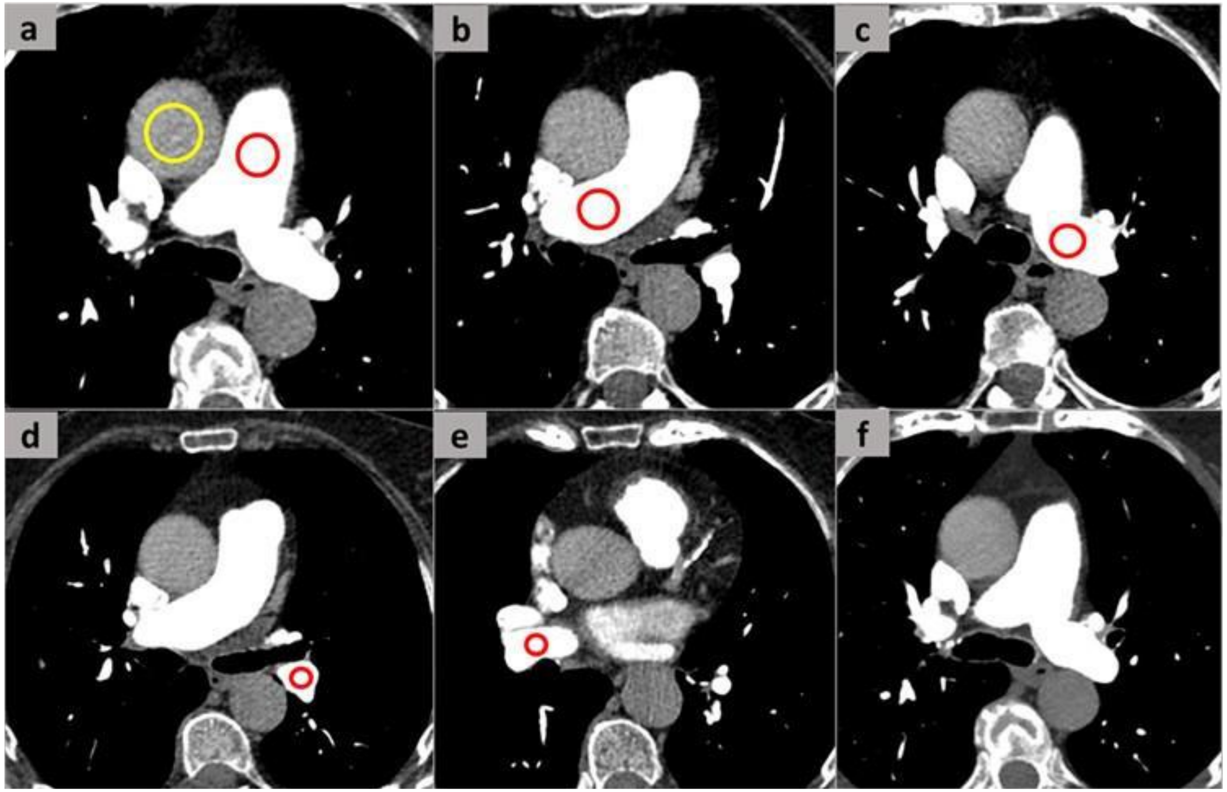

Figure S2. Example of the calculation of normal volumetric iodine density of the right lung. A volume of normal lung tissue is delineated using multiplanar Z-effective, lung and mediastinal windows. By measuring iodine density in the left atrium and taking 50% of this value as a reference, the vascular component is estimated and segmented (in green). The remaining volume corresponds to normal lung tissue (pink coloured) and its iodine density is regarded as the volume iodine density for that lung. The operation is repeated in the left lung, and the mean of both measurements is recorded as the volume iodine density of normal lung in that patient.

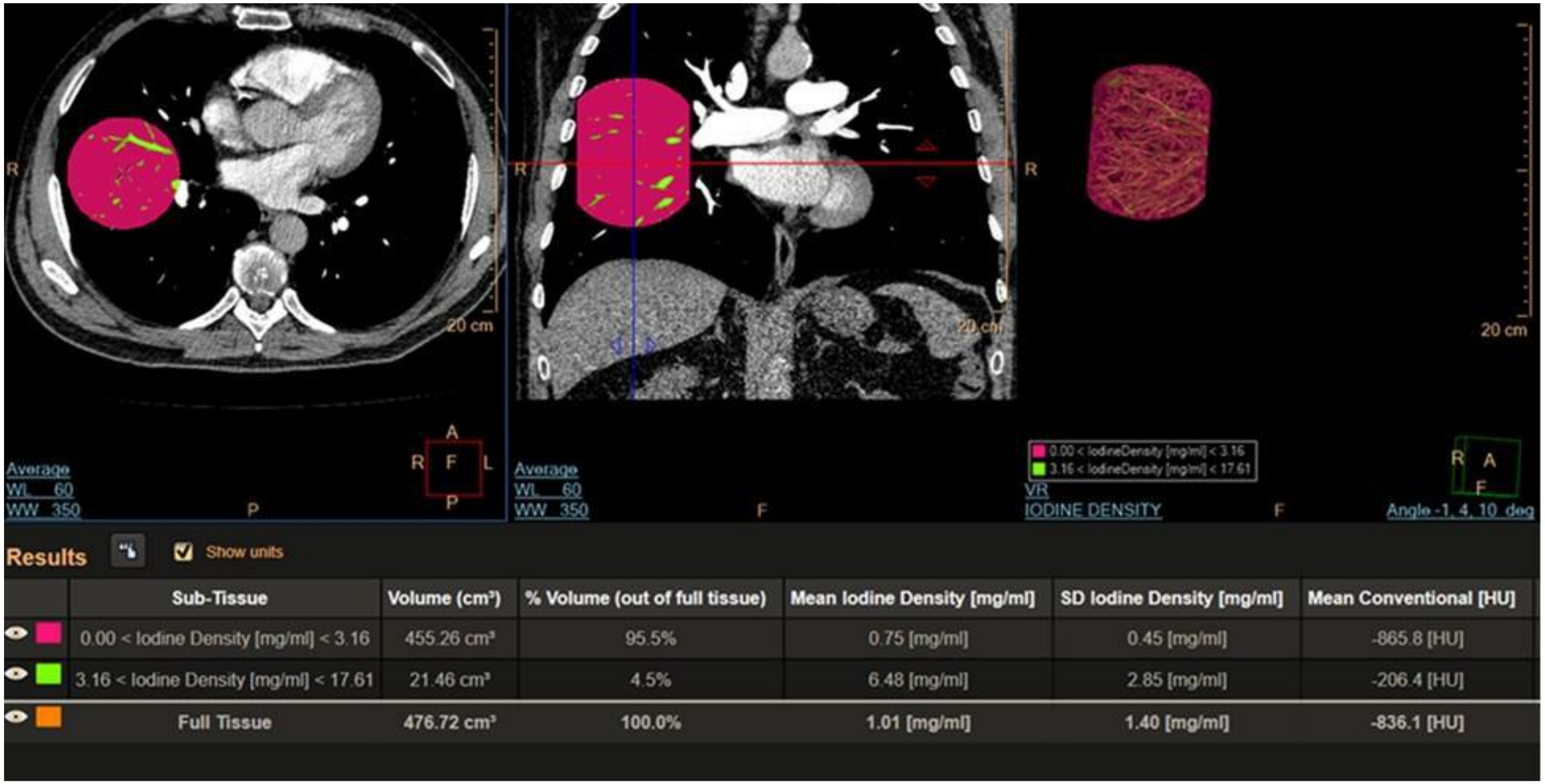

Results ☑ Show units

	Sub-Tissue	Volume (cm³)	% Volume (out of full tissue)	Mean Iodine Density [mg/ml]	SD Iodine Density [mg/ml]	Mean Conventional [HU]
	0.00 < Iodine Density [mg/ml] < 3.16	455.26 cm³	95.5%	0.75 [mg/ml]	0.45 [mg/ml]	-865.8 [HU]
	3.16 < Iodine Density [mg/ml] < 17.61	21.46 cm³	4.5%	6.48 [mg/ml]	2.85 [mg/ml]	-206.4 [HU]
	**Full Tissue**	**476.72 cm³**	**100.0%**	**1.01 [mg/ml]**	**1.40 [mg/ml]**	**-836.1 [HU]**

Figure S3. Example of the calculation of volumetric iodine density of hypoperfused lung in a patient with pulmonary embolism. Using multiplanar Z-effective and iodine density images, pathologic area is delineated. As for normal lung, iodine density in the left atrium is measured and 50% of this value is taken as a reference for the vascular component. Tissue with iodine density below that threshold is considered the lung compartment (in pink), and its iodine density is regarded as the volume iodine density for hypoperfused lung in that case.

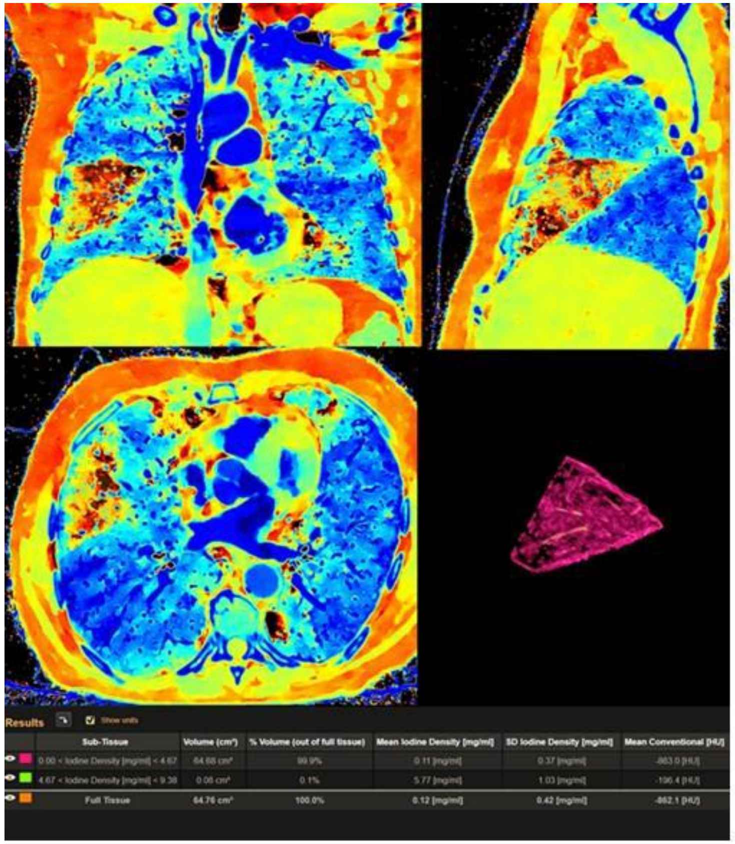

Figure S4. Examples of different grading of quality of Z-effective maps. *a*, excellent, diffusely homogeneous parenchyma; *b*, adequate, some non-significant areas of heterogeneity; *c,* acceptable, with some areas of significant heterogeneity of the map; and *d*, poor quality, with major limitation to evaluate possible areas of hypoperfusion

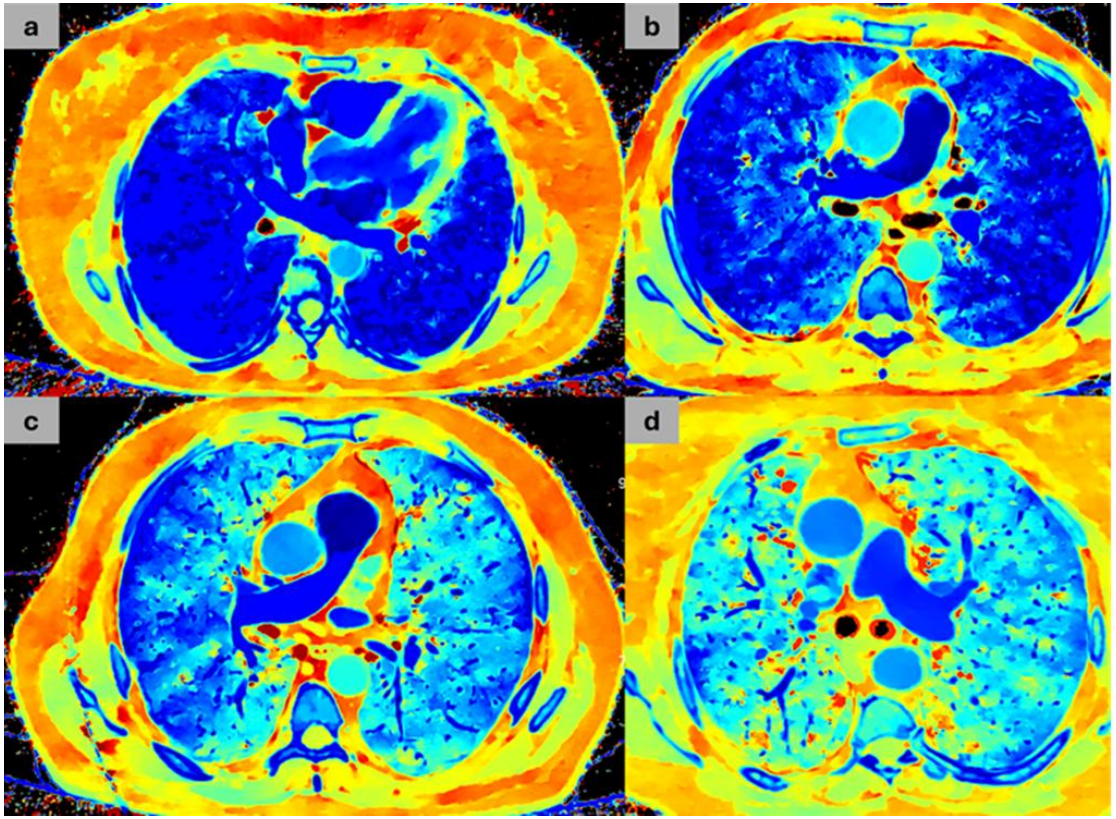

Figure S5. Examples of different grades of artifacts severity in Z-effective maps. *a*, absence of artifacts; *b*, mild that do not interfere; *c*, moderate that slightly affect reading; *d*, moderate that clearly interfere with reading.

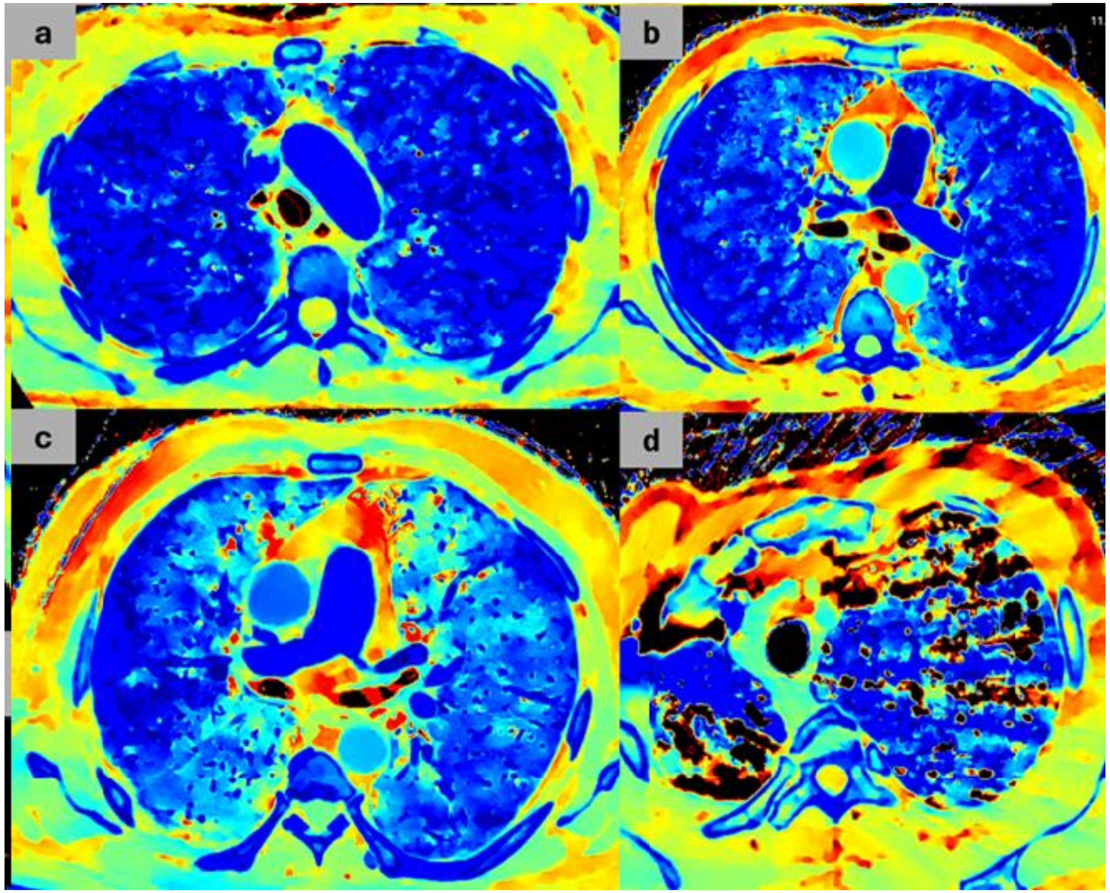

Figure S6. Correlation between body weight and mean pulmonary artery attenuation in conventional images of both readers by protocol.

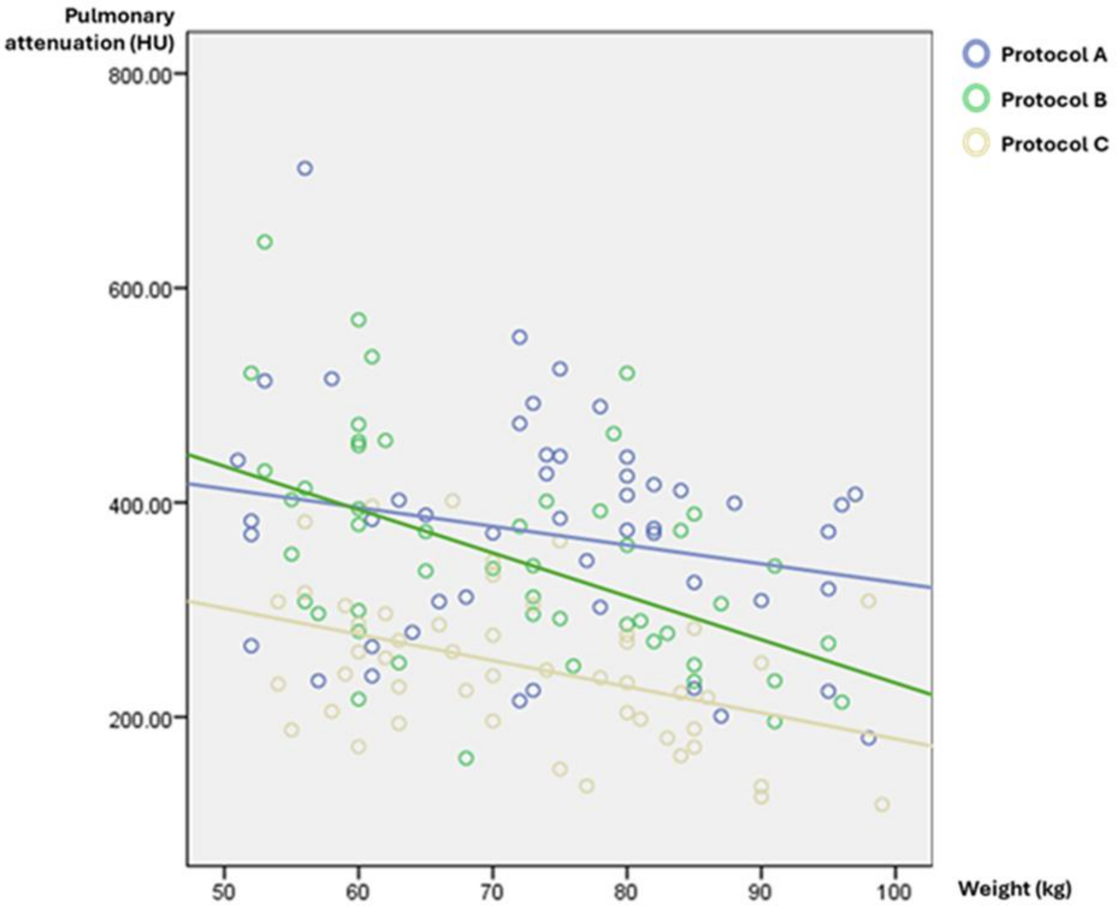

Figure S7. 92-year-old woman with suspected pulmonary embolism due to dyspnea and elevated D-dimer. CT pulmonary angiography at conventional (*a*) and low-energy virtual monoenergetic images (*b*) achieved an attenuation of 107 and 243 HU at the ascending aorta, respectively. Type-B aortic dissection was suspected, and an aortic CT (*c*) was repeated confirming the existence of thrombosed chronic dissection that was subsequently confirmed as a previous diagnosis performed in another center and not reported by the patient.

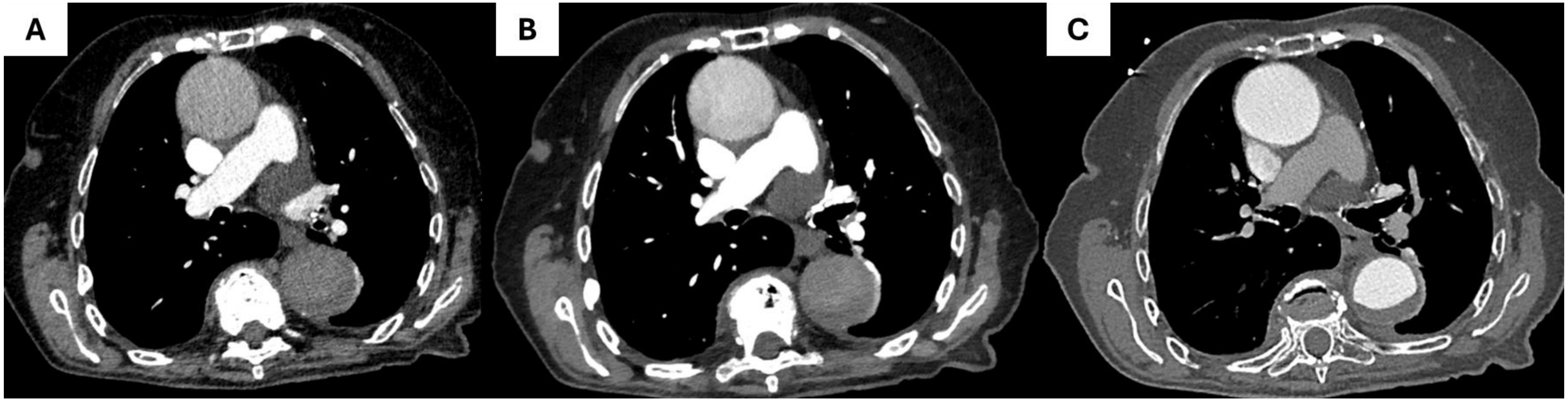

Figure S8. 60-year-old man with previous arterial hypertension and suspected pulmonary embolism due to chest pain and elevated D-dimer. CT pulmonary angiography at conventional (*a*) and low-energy virtual monoenergetic (*b*) images achieved an attenuation of 157 and 400 HU at the ascending aorta, respectively. Low attenuation crescent was seen in the descending aorta, that was confirmed to correspond to high attenuation intramural hematoma in the locator image performed for the bolus tracking (*c*). A non-enhancing and aortic CT were subsequently performed and confirmed the diagnosis. The patient was admitted and managed conservatively. He developed renal insufficiency during the first days in the hospital, which progressively improved.

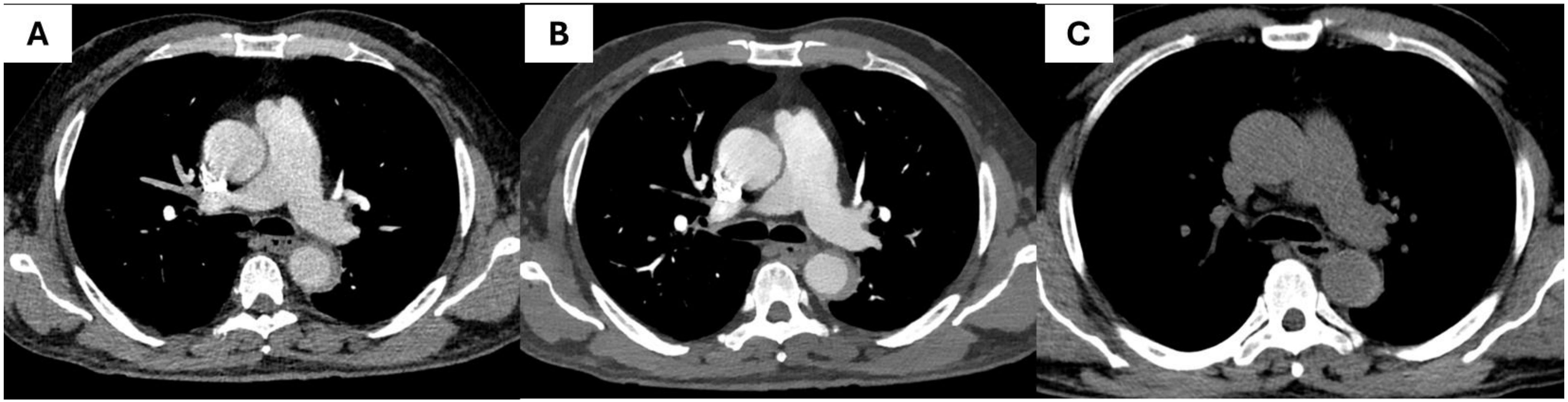

SUPLEMMENTARY TABLES

Table S1. Mean vascular attenuation and minimum values at different pulmonary artery levels at conventional images by protocol.

		PROTOCOL A		PROTOCOL B		PROTOCOL C		*p* values*		
		Mean ± standard deviation	Minimum	Mean ± standard deviation	Minimum	Mean ± standard deviation	Minimum	*p* value A vs B	*p* value A vs C	*p* value B vs C
**Pulmonary trunk**	Reader 1	374.9 ±110.0	178	352.2 ± 104.6	177	249.3 ± 71.9	114	0.461	0.000	0.000
	Reader 2	378.1± 110.3	177	354.3 ± 103.5	181	248.0 ± 69.9	111	0.238	0.000	0.000
**Right pulmonary artery**	Reader 1	359.0 ± 102.3	178	341.4 ± 100.2	157	241.1 ± 69.2	120	0.654	0.000	0.000
	Reader 2	361.0 ± 99.5	177	339.0 ± 98.5	163	239.6 ± 68.4	108	0.227	0.000	0.000
**Left pulmonary artery**	Reader 1	368.5 ± 102.1	178	341.9 ± 98.5	166	240.5 ± 68.1	98	0.332	0.000	0.000
	Reader 2	367.1 ± 101.0	179	340.8 ± 99.5	162	239.8 ± 68.1	98	0.228	0.000	0.000
**Right interlobar artery**	Reader 1	376.6 ± 111.5	187	361.3 ± 111.0	151	245.6 ± 69.8	126	0.728	0.000	0.000
	Reader 2	372.1 ± 109.2	181	360.7 ±111.0	156	244.3 ± 73.6	129	0.362	0.000	0.000
**Left interlobar artery**	Reader 1	387.1 ± 113.1	181	365.7 ± 113.0	157	255.9 ± 75.4	120	0.553	0.000	0.000
	Reader 2	385.9 ± 114.9	184	364.3 ± 113.4	159	252.7 ± 74.6	113	0.387	0.000	0.000

* Overall differences between protocols are statistically significant (p = 0.000) for all measurements

Table S2. Mean vascular attenuation and minimum values at different pulmonary artery levels at low-energy virtual monoenergetic images by protocol.

		**PROTOCOL A**		**PROTOCOL B**		**PROTOCOL C**		***p* values***		
		Mean ± standard deviation	Minimum	Mean ± standard deviation	Minimum	Mean ± standard deviation	Minimum	*p* value A vs B	*p* value A vs C	*p* value B vs C
**Pulmonary trunk**	Reader 1	1056.3 ±370.2	407	956.0 ± 330.6	423	618.2 ± 204.0	243	0.238	0.000	0.000
	Reader 2	1059.8 ± 366.2	427	1011.4 ± 561.4	424	619.3 ± 202.8	240	0.814	0.000	0.000
**Right pulmonary artery**	Reader 1	1075.1 ± 377.5	488	967.2 ± 347.3	415	617.8 ± 203.7	249	0.227	0.000	0.000
	Reader 2	1064.9 ± 370.5	458	968.8 ± 348.9	393	619.5 ± 203.6	251	0.309	0.000	0.000
**Left pulmonary artery**	Reader 1	1060.6 ± 356.7	449	939.9 ± 344.1	412	617.2 ± 203.1	245	0.228	0.000	0.000
	Reader 2	1055.6 ± 358.6	450	961.8 ± 326.5	387	622.0 ± 205.5	254	0.294	0.000	0.000
**Right interlobar artery**	Reader 1	1052.1 ± 357.5	446	967.4 ± 332.1	365	619.6 ± 211.8	267	0.362	0.000	0.000
	Reader 2	1056.2 ± 349.9	447	959.1 ± 356.1	375	618.2 ± 208.1	274	0.366	0.000	0.000
**Left interlobar artery**	Reader 1	1037.9 ± 335.2	483	657.4 ± 343.0	372	620.7 ± 343.0	270	0.387	0.000	0.000
	Reader 2	1035.6 ± 338.6	435	959.6 ± 342.3	371	627.5 ± 206.4	253	0.429	0.000	0.000

* Overall differences between protocols are statistically significant (p = 0.000) for all measurements

Table S3. Distribution of vascular attenuation percentile values at different pulmonary artery levels and in the ascending aorta at conventional images and low-energy virtual monoenergetic images by protocol for reader 1.

		Percentile				
	Protocol	10	25	50	75	90
**Pulmonary trunk**	A	225,0	289,0	368,0	448,5	515,0
	B	219,0	275,5	343,0	408,5	512,0
	C	**156,0**	**192,5**	243,0	300,0	365,0
**Right pulmonary artery**	A	206,0	291,0	367,0	427,0	502,0
	B	229,0	265,0	324,0	400,5	492,0
	C	**157,0**	**188,0**	242,0	283,0	363,0
**Left pulmonary artery**	A	223,0	289,5	380,0	438,0	497,0
	B	235,0	268,5	332,0	399,5	490,0
	C	**148,0**	**194,0**	240,0	283,0	333,0
**Right interlobar artery**	A	222,0	290,0	385,0	433,0	516,0
	B	221,0	286,5	360,0	422,0	537,0
	C	**140,0**	**195,5**	246,0	285,5	328,0
**Left interlobar artery**	A	238,0	303,0	396,0	455,0	514,0
	B	241,0	278,5	347,0	427,0	539,0
	C	**136,0**	**197,5**	254,0	301,0	361,0
**Ascending aorta**	A	101,0	138,5	208,0	263,5	327.0
	B	**81,0**	112,5	173,0	213,0	252.0
	C	**74,0**	120,0	136,0	171,5	210.0
**Pulmonary trunk at LEVMI**	A	563,0	742,5	1075,0	1309,0	1528,0
	B	537,0	742,5	907,0	1105,0	1518,0
	C	363,0	468,0	595,0	757,0	921,0
**Right pulmonary artery at LEVMI**	A	561,0	787,5	1108,0	1298,0	1532,0
	B	575,0	715,5	901,0	1181,5	1521,0
	C	364,0	482,0	582,0	747,5	899,0
**Left pulmonary artery at LEVMI**	A	582,0	789,5	1093,0	1239,0	1541,0
	B	532,0	712,5	867,0	1146,5	1494,0
	C	349,0	473,5	603,0	723,5	882,0
**Right interlobar artery at LEVMI**	A	569,0	770,5	1084,0	1254,5	1460,0
	B	578,0	752,0	928,0	1136,0	1351,0
	C	293,0	475,0	615,0	726,5	900,0
**Left interlobar artery at LEVMI**	A	592,0	759,5	1080,0	1246,5	1400,0
	B	561,0	705,5	895,0	1151,0	1477,0
	C	295,0	456,0	623,0	750,5	889,0
**Ascending aorta at LEVMI**	A	189,0	288,5	547,0	694,5	859.0
	B	153,0	248,0	401,0	520,5	688.0
	C	147,0	269,0	329,0	412,5	512.0

In bold values below 200 HU for pulmonary artery or 100 HU for aortic attenuation.
LEVMI: low-energy virtual monoenergetic images

Table S4. Distribution of vascular attenuation percentile values at different pulmonary artery levels and in the ascending aorta at conventional images and low-energy virtual monoenergetic images by protocol for reader 2.

		Percentile				
	Protocol	10	25	50	75	90
**Pulmonary trunk**	A	230,0	291,0	375,0	446,0	516,0
	B	220,0	277,0	349,0	408,5	513,0
	C	**165,0**	**194,5**	239,0	294,5	340,0
**Right pulmonary artery**	A	212,0	297,0	367,0	421,0	502,0
	B	217,0	270,0	332,0	397,5	498,0
	C	**148,0**	**195,5**	240,0	286,5	324,0
**Left pulmonary artery**	A	228,0	291,0	372,0	432,5	493,0
	B	225,0	266,5	326,0	392,5	492,0
	C	154,0	197,0	238,0	286,0	329,0
**Right interlobar artery**	A	218,0	289,0	385,0	431,5	510,0
	B	237,0	286,5	340,0	418,5	540,0
	C	**144,0**	**194,5**	250,0	286,5	336,0
**Left interlobar artery**	A	234,0	304,0	394,0	447,0	488,0
	B	234,0	273,0	351,0	426,0	538,0
	C	**136,0**	**198,5**	254,0	292,0	341,0
**Ascending aorta**	A	100,0	136,5	201,0	264,0	317,0
	B	**86,0**	116,0	178,0	213,0	263,0
	C	**77,0**	120,5	137,0	175,0	206,0
**Pulmonary trunk at LEVMI**	A	563,0	739,0	1071,0	1310,5	1535,0
	B	536,0	742,0	908,0	1091,5	1537,0
	C	380,0	468,5	592,0	757,5	922,0
**Right pulmonary artery at LEVMI**	A	570,0	763,0	1075,0	1269,0	1525,0
	B	562,0	719,5	888,0	1183,5	1528,0
	C	369,0	474,5	611,0	745,0	930,0
**Left pulmonary artery at LEVMI**	A	574,0	771,5	1088,0	1282,5	1414,0
	B	581,0	747,5	901,0	1147,5	1485,0
	C	370,0	471,5	600,0	730,5	910,0
**Right interlobar artery at LEVMI**	A	569,0	771,0	1134,0	1250,5	1464,0
	B	585,0	750,0	929,0	1137,0	1472,0
	C	300,0	469,5	600,0	741,5	866,0
**Left interlobar artery at LEVMI**	A	570,0	741,0	1094,0	1240,0	1449,0
	B	559,0	719,5	892,0	1202,0	1490,0
	C	331,0	468,5	628,0	740,0	897,0
**Ascending aorta at LEVMI**	A	185,0	289,5	547,0	694,0	886,0
	B	145,0	241,0	401,0	517,0	682,0
	C	149,0	260,5	328,0	418,5	499,0

In bold are values below 200 HU for pulmonary artery or 100 HU for aortic attenuation.

LEVMI: low-energy virtual monoenergetic images

Table S5. Pearson's correlation coefficient of weight and body mass index with mean pulmonary artery attenuation of both readers by protocol.

		**Correlation coefficient (confidence interval 95%)**	***p* value**
**Weight**	Protocol A	-0.219 (-0.471; 0.066)	0.131
	Protocol B	-0.458 (-0.674; -0.236)	0.001
	Protocol C	-0.412 (-0.621; -0.148)	0.003
**Body mass index**	Protocol A	-0.162 (-0.424; 0.125)	0.265
	Protocol B	-0.338 (-0.566; -0.063)	0.017
	Protocol C	-0.237 (-0.468; -0.048)	0.101

Table S6. Number of patients with aortic attenuation below 100 HU at conventional and low-energy virtual monoenergetic images by protocol and by reader.

	**Conventional**		**Low-energy**	
	Reader 1	Reader 2	Reader 1	Reader 2
**Protocol A**	4	3	1	0
**Protocol B**	8	8	1	1
**Protocol C**	8	8	2	2

Table S7. Dose length product and volume CT dose index by protocol.

	**Protocol**	**Median (interquartile range; 25,75)**	***p* value**
**Dose length product**	A	298 (228, 333)	0.111
	B	252 (215, 318)	
	C	261 (223, 213)	
**Volume CT dose index**	A	6.5 (5.4, 6.8)	0.177
	B	5.7 (5.0, 7.2)	
	C	6.1 (5.1, 6.8)	

Dose length product in mGy; volume CT dose index in mGY.cm

Table S8. Spearman's rank correlation of weight and body mass index with dose length product and volume CT dose index.

	**Spearman's rank correlation (confidence interval 95%)**	***p* value**
**Dose length product**		
**Weight**	0.810 (0.745-0.860)	0.000
**Body mass index**	0.607 (0.491-0.702)	0.000
**Volume CT dose index**		
**Weight**	0.798 (0.729-0.851)	0.000
**Body mass index**	0.655 (0550-0.740)	0.000
